# A Lean Quality Improvement Initiative to Enhance Tobacco Use Treatment in a Cancer Hospital

**DOI:** 10.3390/ijerph17062165

**Published:** 2020-03-24

**Authors:** Colleen Meyer, Sara Mitra, Ellen Ruebush, Laurel Sisler, Kyle Wang, Adam O. Goldstein

**Affiliations:** 1 Department of Family Medicine, The University of North Carolina at Chapel Hill, Chapel Hill, NC 27759, USA; laurel_sisler@med.unc.edu (L.S.); adam_goldstein@med.unc.edu (A.O.G.); 2 Lineberger Comprehensive Cancer Center, The University of North Carolina at Chapel Hill, Chapel Hill, NC 27599, USA; saramitra@unc.edu (S.M.); kyle_wang@med.unc.edu (K.W.)

**Keywords:** tobacco use, smoking cessation, cancer patients, process improvement

## Abstract

Sustained tobacco use after cancer diagnosis decreases treatment effectiveness while increasing treatment side effects, primary cancer recurrence, and the occurrence of secondary cancers. Delivering tobacco use treatment to fewer patients due to inefficient workflow represents missed opportunities to deliver life-saving care. In 2017, the National Cancer Institute initiated the Cancer Cessation Initiative (C3I) to push new tobacco cessation resources into cancer centers across the United States. This grant allowed the University of North Carolina Tobacco Treatment Program (UNC TTP) to dramatically expand tobacco use treatment (TUT) services to patients at the North Carolina Cancer Hospital (NCCH). With this push, the team saw an opportunity to utilize Lean Six Sigma, a set of quality improvement (QI) tools, to streamline their processes and uncover the root causes of program inefficiencies. A 12-month QI project using the Lean A3 problem-solving tool was implemented to examine the team’s workflow. The study team mapped out the processes and, as a result, developed multiple “experiments” to test within the NCCH to address workflow efficiency and clinical reach. Outcome measures from the baseline to follow-up included: (1) the number of new patient referrals per month, and (2) the number of counseling sessions delivered per month. From the baseline to final state, the team’s referrals increased from a mean of 10 to 24 per month, and counseling sessions increased from a mean of 74 to 84 per month. This project provided a deeper understanding of how workflow inefficiencies can be eliminated in the clinical setting, how technology can be harnessed to increase reach, and finally, that soliciting and using feedback from NCCH leadership can remove barriers and improve patient care.

## 1. Introduction

Tobacco use remains one of the nation’s most significant public health threats and is estimated to be responsible for over 400,000 deaths in the United States every year [1]. Additionally, most patients receiving cancer treatment use tobacco or have a history of using tobacco [2]. Tobacco use accounts for approximately 30% of all cancer deaths and 80% of lung cancer deaths [1]. While improvements in cancer treatment have increased survivorship, continued tobacco use after cancer diagnosis decreases treatment efficacy, increases treatment side effects, and can lead to a decrease in patient-reported quality of life [2].

Treating tobacco use is an essential component of cancer care; patients that continue to use tobacco risk development of secondary cancers, inferior treatment outcomes, and exacerbation of co-morbid health conditions [3]. After a cancer diagnosis, many patients have increased motivation to become tobacco-free, but the oncology community has struggled to provide reliable, effective smoking cessation treatment [4]. Physician-identified barriers to providing cessation support are recognized in the literature and include lack of time, experience, and resources in the oncology setting [5]. Research is limited on best practices for delivering tobacco use treatment (TUT) to oncology patients at a large medical center.

In 2010, the University of North Carolina created the Tobacco Treatment Program (UNC TTP) to address this critical health challenge. This multidisciplinary team is dedicated to providing evidence-based tobacco use treatment to patients [6]. The embedded team at the North Carolina Cancer Hospital (NCCH) is composed of two trained tobacco treatment specialists (TTS) and one research specialist. The TTS work with patients individually to identify and achieve tobacco cessation goals before, during, and after cancer treatment. Beginning in 2017, as a push to provide more cessation resources to the oncology population, the National Cancer Institute (NCI) awarded grants to 42 NCI-designated cancer centers across the country as part of the Cancer Cessation Initiative (C3I) [4]. UNC TTP capitalized on this opportunity by examining ways to improve tobacco cessation treatment for cancer patients at NCCH using a quality improvement (QI) strategy called Lean Six Sigma. Lean is a QI strategy initially developed by the Toyota Motor Company to improve problems related to workflow and quality, while also increasing value to customers. Lean strategies such as “A3 thinking” provide a systematic process where challenges and solutions must follow a rigorous nine-step problem-solving process. Such strategies have been applied in the healthcare setting to improve the quality of patient care delivered by healthcare organizations [7,8].

In this article, we describe a 12-month Lean initiative designed to improve workflow efficiency and patient care. 

## 2. Materials and Methods

### 2.1. Setting

The QI project was initiated in September 2018 at NCCH with an existing, embedded tobacco treatment program (TTP). The QI team consisted of the two TTS and the research specialist from the NCCH tobacco treatment program, two additional TTS who worked within the larger hospital system, a clinical social worker, and a nurse navigator from the otolaryngology (ENT) oncology clinic. The two TTS based at NCCH were the only clinicians delivering tobacco treatment to cancer patients throughout the QI initiative. A trained Lean coach from the UNC Department of Family Medicine guided the team through a series of monthly trainings to apply Lean concepts to this project. The team also went through a series of all-day trainings in Lean Principles to ensure the concepts were well-understood before applying them in the clinical setting.

### 2.2. Application of the Lean QI Tools

The QI team focused on the Lean A3 problem-solving tool for this project. A3 problem-solving is a systematic, nine-step process that helps structure a project from conception to completion (Appendix A). First, the team created a “Reason for Action” by developing a problem and importance statement, as well as refining the scope of the project. Next, the team mapped the “Current State” and future “Target States” related to workflow and patient care. The team selected two metrics to measure throughout the project—new patient referrals and counseling sessions. At the current state, the program received an average of 10 new patient referrals per month and conducted an average of 74 counseling sessions per month. A “Gap Analysis” was completed next, which is a Lean strategy used to identify the root causes behind gaps between the current and target states. These potential root causes were generated using the “Five Whys,” a process that involves asking why the gap exists five consecutive times to understand the root of an inefficiency. For this project, the team identified eight potential gaps between current and target states, as well as corresponding root causes for each gap. For practicality, the team selected four gaps to focus on during this QI project: (1) low program awareness, (2) inconsistent referral process, (3) a low referral rate from a strongly affiliated department (Radiation Oncology), and (4) burdensome data collection. Then, using the “If, Then” solution approach, the team generated possible solutions with corresponding outcomes. Outcome measures were the number of newly referred patients and the number of counseling sessions per month. The team felt these measures reflected the project goals of improved workflow efficiency and increased patient care. Next, the team worked together to brainstorm potential experiments that could help achieve the project goals of improved workflow efficiency and increased patient care (see Appendix A for details).

### 2.3. Lean Experiments

Lean experiments are described as rapid improvement activities because they are testing the impact of incremental changes to a process to determine if there is even a slight impact on outcomes. The team conducted four “Rapid Experiments” (Table 1) thought to have the greatest impact on the identified gaps within the project timeline. 

A TTS and the ENT nurse navigator from the QI team attended a tumor board meeting for each oncology department, which is the main gathering of providers to plan treatments and share information. The purpose of attending these meetings was twofold—to provide attendees with general information about TTP, and to educate providers on how to refer new patients to the program using a standard referral protocol through the Electronic Health Record (EHR). These experiments (1 and 2 in Table 1) were designed to increase new patient referrals, as well as streamline the mechanism through which new patients are referred for TUT. Prior to the QI project, patients were being referred to TTP via several inconsistent referral methods, including emails, in-the-moment pages, phone calls, and via the EHR. Consequently, TTS were navigating an inefficient referral workflow and spending valuable time processing referrals from multiple sources, rather than providing counseling to patients. By implementing one standard referral protocol, the QI team anticipated a positive effect on workflow efficiency, providing TTS with more time to complete counseling sessions.

**Figure 1:**
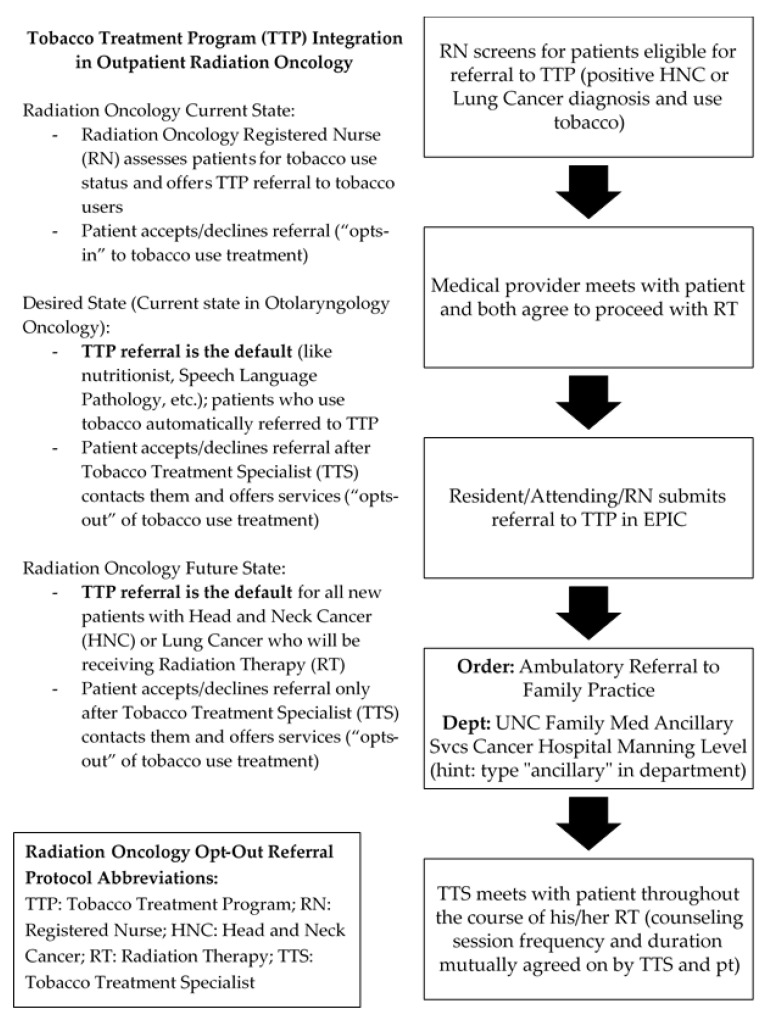
Radiation oncology opt-out referral protocol.

The third experiment conducted by the QI team (3 in Table 1) involved replicating an opt-out protocol modeled on the current workflow employed in the ENT oncology department. The ENT oncology department operates from an opt-out framework, in which eligible patients are automatically referred for TUT unless explicitly declined by the patient. As a result, TTP has historically experienced a high number of new patient referrals from this clinic and has enjoyed a close working relationship with providers from this department. For this experiment, the QI team set out to replicate this opt-out model in Radiation Oncology (Figure 1), another department with which TTP has historically had a close professional relationship. Because of this experiment, the team anticipated a positive effect on new patient referrals, contributing to the goal of increased patient care.

The final experiment entailed a member of the QI team meeting with TTP leadership to discuss reduction of the data collection burden (4 in Table 1). In the prior state, TTS were collecting data and reporting on four metrics on a monthly basis: the number of unique patients reached, number of unique patients attempted, number of counseling sessions, and six-month quit rate. A member of the QI team met with the leadership team, and all were able to agree on reducing the data collection burden. Post-experiment, TTS were responsible for tracking only one metric—the number of unique patients reached per month. Reducing the data collection load provided TTS with more time to counsel patients.

## 3. Results

**Figure 2:**
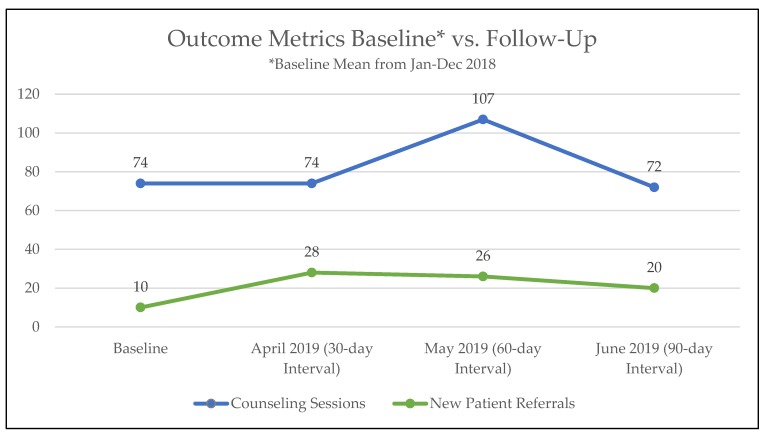
Outcome metrics comparing baseline to follow-up at 30-, 60-, and 90-day intervals.

The QI team initiated project planning in July 2018. In accordance with Lean principles, baseline data was established by averaging the number of new referrals and counseling sessions from a 12-month period of January to December 2018. During January and February 2019, the team engaged in planning the QI experiments and experiments began in March 2019. Post-experiment data collection was conducted from April through June 2019 to track 30-, 60-, and 90-day outcomes (Figure 2). Had the project continued past the 90-day follow-up interval, a new round of experiment planning and implementation would have occurred. All data was extracted from the EHR via a charting tool that the team uses regularly for documenting, called a flowsheet. From January through June 2019, 1527 patients were eligible for tobacco use treatment at NCCH, an average of 254 patients per month. 

As shown in Figure 2, mean new patient referrals increased by 140%, and mean counseling sessions increased by 13%. Before the initiation of the project, TTP received at baseline a mean of 10 new patient referrals per month that increased to a mean of 24 per month in the follow-up periods. After each of the 30-, 60-, and 90-day follow-up intervals, new patient referrals increased. Counseling sessions also increased from a mean of 74 at baseline to 84 at the follow-up periods. However, counseling session changes at the 30- 60-, and 90-day time periods were more variable, with a large increase only at the 60-day follow-up period. 

## 4. Discussion

Tobacco use treatment for oncology patients is an important and necessary component to the overall cancer care experience. However, scarce research exists regarding effective and efficient delivery of this treatment to a large number of patients. At the North Carolina Cancer Hospital, an embedded tobacco treatment program saw an opportunity to improve the efficiency of service delivery to patients. In this article, we described the development and implementation of a QI project based on Lean Six Sigma principles, which generated several insights. Most importantly perhaps, more patients received some degree of TUT counseling. More patients receiving cessation interventions should lead to more patients becoming tobacco-free [3]. 

This QI project has important practical implications for providers and healthcare systems that want to improve cessation counseling in cancer care settings. First, while the QI project led to increased mean patient referrals and counseling sessions per month from baseline to follow-up, the actual picture was more complicated. The mean number of new patient referrals and counseling sessions certainly increased from baseline to follow-up, but referrals increased significantly more than actual sessions. Referrals alone may not always result in increased counseling, as some of these referrals may not lead to visits. It also appeared that decreases in progress may have occurred at the 90-day post-intervention follow-up point in new referrals and counseling sessions, indicating a need for additional strategies to maintain results beyond short-term gains.

Second, as Lean is a commitment to continuous improvement and knows no finish line [7], we were able to identify several pitfalls that occurred. For instance, as more new patients were referred to the program, TTS had to conduct more initial assessments, which were more in-depth and time-consuming. Oncology providers were responding to the experiments and referring new patients, but with each new patient referred, TTS had to spend additional time enrolling the patient in the program. Yet, the labor to handle the increased referrals remained constant with two NCCH-based TTS, resulting in insufficient time to take on larger increases in volume. The spike in counseling sessions seen in the 60-day follow-up period could also reflect inconsistent distribution of monthly work responsibilities outside of the clinics. As the TTS are responsible for more duties than counseling, it is possible that the month of May provided the TTS with additional work time to complete counseling sessions, but April and June pulled the TTS towards other commitments and work responsibilities.

Third, QI projects can also show how workflow inefficiencies can be reduced within clinical settings. Our team found this to be true through the experiment of TTS attending tumor board meetings and educating oncology providers on how to refer patients correctly through the EHR. Using the EHR as the primary mechanism to refer new patients improved the team’s efficiency, as TTS could readily identify referrals and contact them by telephone to initiate tobacco cessation counseling. By using the EHR for referrals, the TTS no longer had to spend time physically locating patients within oncology clinics, a process that usually involved significant wastes of time by tracking, locating, and subsequently waiting to meet with patients amid their other medical appointment(s). Additionally, educating providers on the correct referral protocol reduced the time spent by TTS amending referrals submitted incorrectly within the EHR.

Fourth, stakeholder feedback and leadership engagement can assist with the removal of barriers that hinder provision of patient care. Through successful stakeholder engagement, our QI team was able to successfully reduce the amount of burdensome data collected. Less time spent by TTS collecting, recording, and analyzing data resulted in more time to reach new patients and to make attempts to follow-up with current patients.

Fifth, to sustain progress, technology must be harnessed to increase visibility and contact with eligible patients. Because of funding from NCI, the program was charged with reaching more than 90% of cancer patients eligible for tobacco use treatment. In this project, the QI team realized that this goal was unattainable with the current staff of two full-time TTS. Telephonic outreach is a proven effective and efficient mode of delivering tobacco use treatment [9]. Incorporating technology, such as an automated digital platform to contact and schedule patients, is the next logical step toward achieving this mission in the most efficient and cost-effective manner [10]. In addition, relying on humans to sustain gains requires ongoing time and effort that is not always feasible [11]. We are now implementing solutions, such as enabling auto-referrals in the EHR, to decrease reliance on oncology providers to refer patients; this will decrease the need for continued outreach on the part of members of the QI team [12,13].

Several factors limited the interpretation of our findings. This project was conducted at an academic medical institution by an already established tobacco treatment program, so the results may not be generalizable. However, we believe that both newly created, as well as mature tobacco treatment programs can reproduce these experiments. The QI initiative required a low direct cost to the program but incurred high investments of staff time. While the initial outcomes improved, 90-day gains were limited by TTS’ available clinical time to manage new referrals and complete counseling sessions. Small changes did affect the program, but more significant changes would be necessary to sustain long-term improvements. During the project, the team also elected not to address certain root causes or complete additional experiments due to limitations of time and resources. These more intensive experiments could have yielded results that would have had a more sustained impact than the less intensive experiments the team was able to complete. Should the team decide to revisit the strategies implemented during this project, or initiate new experiments, then another improvement cycle would begin with these experiments. Even with these limitations, this project’s major strength was using Lean principles. Lean allows for the variance of settings by empowering on-the-ground workers to develop solutions unique to their circumstances.

Delivering tobacco use treatment in a more efficient manner remains clinically important. When oncology patients become tobacco-free, benefits seen include decreased side effects, a lower recurrence of primary cancers, and decreased risk of development of secondary cancers [2,3,8]. Lean tools and strategies increased the number of patients in this high-risk group that were referred for evidence-based tobacco use treatment [8]. 

## 5. Conclusions

This article demonstrates that the Lean methodology can help tobacco treatment programs increase delivery of tobacco use treatment to eligible cancer patients [14]. By employing Lean tools, this team was able to generate a positive effect on the number of new patients referred for services, as well as the amount of counseling sessions delivered to active participants. More research is necessary to explore effective means of delivering and sustaining tobacco use treatment to oncology patients on a larger scale [15]. 

## Figures and Tables

**Table 1:** Quality improvement experiments from gap analysis to project goals.

Gap	Experiment	Rationale for Experiment	Outcome Metric Impacted	Project Goal
Oncology providers lack awareness of TTP	(1) TTS will attend oncology tumor board meetings to present about the program and services	Oncology providers will have improved awareness of the program	New patient referrals	Increased patient care
Oncology providers are unfamiliar with how to refer new patients to TTP	(2) TTS will attend oncology tumor board meetings to provide education on how to refer new patients via the Electronic Health Record	New patients will be referred using one standard protocol, which will allow TTS more time to counsel patients each month	New patient referrals & counseling sessions	Increased patient care and improved workflow efficiency
Low referral rate from Radiation Oncology department	(3) Radiation Oncology department will implement an opt-out protocol based on ENT department model	Implementing an opt-out protocol will increase the number of new patients referred for TUT	New patient referrals	Increased patient care
Too much time spent by TTS collecting burdensome data	(4) TTS will meet with program leadership to agree on a reduced data collection burden	Less time spent by TTS collecting, recording, and analyzing data will allow for more time to counsel patients each month	Counseling sessions	Improved workflow efficiency

**Figure A1:**
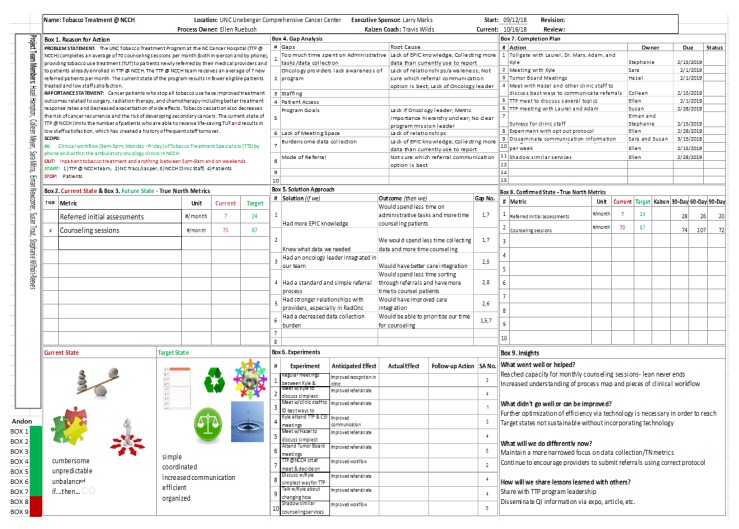
The Lean A3 problem-solving tool demonstrated in this quality improvement initiative. The figure shows the nine steps, or boxes, used in A3 to design, execute, and evaluate the planned project. Abbreviations: TTP, Tobacco Treatment Program; NCCH, North Carolina Cancer Hospital; TUT, tobacco use treatment; TTS, Tobacco Treatment Specialist; RadOnc, Outpatient Radiation Oncology; C3I, Cancer Cessation Initiative; TN, true north; QI, quality improvement.
